# Items

**Published:** 1881-11

**Authors:** 


### ITEMS.

BELIEF is not in our power, but truthfulness is.

DIVINE vengeance comes with feet of lead, but strikes with hands of iron

WHEN a person is struck by lightning, buckets of water should be poured upon his head.

HE needs no other rosary whose thread of life is strung with beads of love and thought.

To purify wells, etc., shower water down them until a candle can burn with a clear flame.

IT is alleged that the oysters found off the Texan coast are the largest and best in the world.

HE who, with good health, has a true friend, may laugh adversity to scorn and defy the world.

TRUE benevolence is to love all men. Recompense injury with justice, and kindness with kindness.

THIS is the present reward of virtuous conduct—that no unlucky consequence can oblige us to regret it.

FLOWERS sweeten the air, rejoice the eye, link us with nature and innocence, and are something to love.

IF the poison of a living serpent is extracted from its fang, in two days it will be found as highly charged as ever with venom.

NO man ought to complain if the world measures him as he measures others. To measure one with his own yardstick may be hard, but it is fair.

A PAPER, on which was written "Mrs. Sophia Loper is my heiress," with a date and signature, was admitted to probate as a will in New Orleans.

OSCAR WILDE, the new English poet, speaks of "unkissed kisses." The trouble with Oscar is that his poems are made up of unthunk thoughts.—*Atlanta Constitution.*

"MALARIA!" said the Old Orchard Beach landlady; "well, no, we haven't got it; folks hain't asked for it, but we'll get it for your family."—*Lake Pleasant News.*

FENDERSON says he has lots of spare time on his hands since he began to attend exclusively to his own business. Formerly he was the hardest worked man in the city.—*Boston Transcript.*

AN "Indian idol" was recently ploughed up in Woodbury, and they don't know whether it's a god or a devil. If it has a handle and holds about two gallons, it's a Woodbury god.—*Canaan News.*

BROTHERHOOD is the term in common use to imply the spirit we should cherish and the conduct we should practice toward our fellow-men, showing how strong is the sense of what the relation ought to involve.

JINKS' parents live up in Buffalo and have to send him money every month to keep his landlord from evicting him. Jinks, in writing back his gratitude, always begins, "My Dear Payrents." *Louisville Courier-Journal.*

A FRENCH surgeon mitigates pain by administrating a series of wave sounds to the affected part by means of a tuning fork and a sounding board. Neuralgia is cured speedily. The vibration is kept up by an electro magnet.

In Africa Major Piute, the Portuguese explorer, discovered a large ant called the Guisonde, that puts the elephant to flight, it is so belligerent, indefatigable and tough. It is of light chestnut color. It draws blood at a bite.

Professor Bell claims that he has succeeded in inventing a machine that will "locate a bullet in the human body." He needn't think that's anything new. Almost every man in Denver totes such an instrument.—*Boston Post.*

A cure for hydrophobia is reported to have been found in a plant found in Tonquin—the hoang-nan. It is a poison something like strychnine, and the patient's organization thus becomes the battle-ground between poison and virus.

Traveler—"How do you brokers manage to undersell the railroad companies?" Scalper—"Vell, you see, ve don't got so much expenses. Dose railroad fellers haf to keep up the rollin' stock an' pay ze hands. We don't. It's all clear profit wit us!"

A good-looking old German, with long hair, sat down, or rather up, in the barber's chair, and was asked whether he would have his hair shingled. He replied: "Mien Kott no! I vant some hair koot off. Vot voot yoa put zum shingles on it pecause?"

